# Validation of Fully Automated VMAT Plan Generation for Library-Based Plan-of-the-Day Cervical Cancer Radiotherapy

**DOI:** 10.1371/journal.pone.0169202

**Published:** 2016-12-29

**Authors:** Abdul Wahab M. Sharfo, Sebastiaan Breedveld, Peter W. J. Voet, Sabrina T. Heijkoop, Jan-Willem M. Mens, Mischa S. Hoogeman, Ben J. M. Heijmen

**Affiliations:** Department of Radiation Oncology, Erasmus MC Cancer Institute, Rotterdam, The Netherlands; Northwestern University Feinberg School of Medicine, UNITED STATES

## Abstract

**Purpose:**

To develop and validate fully automated generation of VMAT plan-libraries for plan-of-the-day adaptive radiotherapy in locally-advanced cervical cancer.

**Material and Methods:**

Our framework for fully automated treatment plan generation (Erasmus-iCycle) was adapted to create dual-arc VMAT treatment plan libraries for cervical cancer patients. For each of 34 patients, automatically generated VMAT plans (autoVMAT) were compared to manually generated, clinically delivered 9-beam IMRT plans (CLINICAL), and to dual-arc VMAT plans generated manually by an expert planner (manVMAT). Furthermore, all plans were benchmarked against 20-beam equi-angular IMRT plans (autoIMRT). For all plans, a PTV coverage of 99.5% by at least 95% of the prescribed dose (46 Gy) had the highest planning priority, followed by minimization of V_45Gy_ for small bowel (SB). Other OARs considered were bladder, rectum, and sigmoid.

**Results:**

All plans had a highly similar PTV coverage, within the clinical constraints (above). After plan normalizations for exactly equal median PTV doses in corresponding plans, all evaluated OAR parameters in autoVMAT plans were on average lower than in the CLINICAL plans with an average reduction in SB V_45Gy_ of 34.6% (*p*<0.001). For 41/44 autoVMAT plans, SB V_45Gy_ was lower than for manVMAT (*p*<0.001, average reduction 30.3%), while SB V_15Gy_ increased by 2.3% (*p* = 0.011). AutoIMRT reduced SB V_45Gy_ by another 2.7% compared to autoVMAT, while also resulting in a 9.0% reduction in SB V_15Gy_ (*p*<0.001), but with a prolonged delivery time. Differences between manVMAT and autoVMAT in bladder, rectal and sigmoid doses were ≤ 1%. Improvements in SB dose delivery with autoVMAT instead of manVMAT were higher for empty bladder PTVs compared to full bladder PTVs, due to differences in concavity of the PTVs.

**Conclusions:**

Quality of automatically generated VMAT plans was superior to manually generated plans. Automatic VMAT plan generation for cervical cancer has been implemented in our clinical routine. Due to the achieved workload reduction, extension of plan libraries has become feasible.

## Introduction

There is a growing evidence that more conformal dose distributions can limit frequently observed side effects in external beam radiotherapy (EBRT) of cervical cancer patients [1,2]. The concavely shaped planning target volume (PTV) of locally-advanced cervical cancer patients requires intensity modulated radiotherapy (IMRT) or volumetric modulated arc therapy (VMAT) techniques in order to improve conformality and subsequent sparing of surrounding healthy tissues compared to 3-dimension conformal radiotherapy (3DCRT) [3,4].

The use of highly conformal dose distributions for cervical cancer is challenged by the large inter-fraction variability in the shape and position of the target volume [5]. To avoid the need for large margins to compensate for the geometrical uncertainties [6], our institute has implemented a plan-of-the-day (PotD) strategy using individualized libraries of treatment plans created in the treatment preparation phase [6,7]. During the daily treatment fractions, a CBCT scan is acquired prior to dose delivery to select the plan from the library that best fits the patient’s internal anatomy. The library is constructed using two planning CTs made with a full and an empty bladder to construct Internal Target Volumes (ITVs) encompassing the cervix-uterus motion for subranges in bladder volume. Up to recently, the library contained two 9-beam IMRT plans (one for empty-to-half-full and one for half-full-to-full bladders) for patients with a uterine fundus displacement between the full and empty bladder CT scan larger than 2.5 cm (large movers). For patients with smaller fundus mobility (small movers), the library contained one 9-beam IMRT plan covering all bladder-induced cervix-uterus motion. Apart from the IMRT plans, a 4-field motion-robust 3DCRT plan was always added, to be selected in fractions with inadequate target coverage by the IMRT plans, or in case of poor CBCT image quality prohibiting proper evaluation of the patient’s anatomy (see for full details [7]). All plans were generated manually.

A library-based PotD approach with multiple pre-treatment generated plans per patient requires an enhanced planning effort. The generally applied, manual trial-and-error process for treatment plan generation is time consuming, and the outcome does largely depend on the efforts and skills of the planner and on allotted planning time. This limits the widespread clinical implementation of library-based plan-of-the-day strategies or puts constraints on the number of plans in a library. In previous studies, we have demonstrated for head and neck and prostate cancer that automated treatment plan generation could substantially reduce the workload compared to conventional planning with equal or superior plan quality [8,9]. The purpose of this work was to develop and validate automatic dual-arc VMAT treatment planning for cervical cancer patients, based on our in-house platform for automated plan generation. For each automated plan generation, a manually contoured planning CT-scan was used as input. For validation of plan quality, fully automatically generated dual-arc VMAT plans (autoVMAT) were compared with i) manually generated, clinical 9-beam IMRT plans (CLINICAL), ii) dual-arc VMAT plans, manually generated by an expert planner (manVMAT), and iii) automatically generated 20-beam equi-angular IMRT plans (autoIMRT). For the large movers, separate analyses were performed for the plans generated for the empty-to-half-full-bladder PTVs and the half-full-to-full-bladder PTVs.

All final plans were generated with our clinical TPS, with an implemented Monte Carlo dose calculation engine. For a subgroup of patients, automatically generated VMAT and IMRT plans were executed at a treatment machine equipped with a novel, fast multi-leaf collimator for treatment time assessment and comparison.

## Material and Methods

### Patients

In this study, the CT- and treatment plan data of 34 patients without pelvic or para-aortic nodal involvement was used to validate multi-criterial autoVMAT planning. All patient-related information was fully anonymized prior conducting the research. According to the regulations of the Ethics Committee of Erasmus MC no ethical approval for this retrospective study was needed as there was no impact on treatment and the applied patient data. EBRT was delivered to a dose of 46 Gy in 2 Gy daily fractions, followed by brachytherapy. Thirty-one patients were treated in prone position, lying on a small bowel displacement system (belly board), and three patients were treated supine. To limit the dose to healthy tissues, all patients were treated according to the plan-of-the-day protocol with 9-beam IMRT plans, as briefly described in the Introduction section and extensively explained in [6,7]. For 24 patients, the library contained a single IMRT plan (small movers, see Introduction), while the other 10 patients had two IMRT plans (for empty-to-half-full and half-full-to-full bladder filling, respectively; large movers), resulting in a total of 44 9-beam IMRT plans. Apart from the 34 patients used for validation of automatic planning, 5 different training patients were used for the wish-list generation (below).

All 44 clinical plans were produced with the clinical TPS (Monaco version 3.3, Elekta AB, Sweden) for a photon beam energy of 10MV, using an Elekta Synergy linear accelerator equipped with the MLCi2 multi-leaf collimator (MLC).

### Plan generations

For each of the 44 clinically applied structure sets in the 34 planning CT-scans, a dual-arc VMAT plan was automatically generated (autoVMAT) and compared with the clinically delivered 9-beam IMRT plan (CLINICAL), and a manually generated dual-arc VMAT plan (manVMAT). Finally, the autoVMAT plan was also benchmarked against an automatically generated 20-beam equi-angular IMRT plan, delivered with dynamic multi-leaf collimation (autoIMRT) (technical details below).

All clinical and study plans were generated in line with our clinical PotD protocol. To establish the planning target volume (PTV), the cervix-uterus ITV (see Introduction), nodal CTV and parametria were combined and expanded by a 1 cm margin in all directions. At least 99.5% of the PTV should receive ≥ 95% of the prescribed dose (D^P^), and no more than 0.2% of the PTV may receive ≥ 110% of the D^P^. Dose distributions should be conformal while maximally sparing the organs at risk (small bowel (SB), rectum, bladder, and sigmoid), giving highest priority to SB sparing [10]. As in clinical practice, for all 44 structure sets, autoVMAT, manVMAT and autoIMRT plans were produced for an Elekta Synergy linear accelerator equipped with the MLCi2 multi-leaf collimator (MLC) and a photon beam energy of 10MV. For treatment time studies, for a subgroup of 5 patients, automatic plans were also created for the much faster Agility MLC (Elekta AB, Sweden) with 160 leaves, with effective leaf speeds of up to 6.5 cm/sec [11,12].

### ClinicaL IMRT plan generation

The clinically applied 9-beam, step-and-shoot IMRT plans were manually generated by dosimetrists in Monaco, using pre-optimized beam angles established with Erasmus-iCycle [13].

### Manual generation of VMAT plans (manVMAT)

For each planning CT scan/structure set, a dual-arc VMAT plan (manVMAT) was generated manually with the clinical TPS in a trial-and-error process by an expert planner (AWMS), in the absence of time pressure and without prior knowledge of results of other planning strategies.

### Automated generation of VMAT and 20-beam IMRT plans (autoVMAT, autoIMRT)

In a previous study [14], our in-house Erasmus-iCycle optimizer was prepared for fully automated multi-criteria generation of treatment plans for cervical cancer patients. These were non-deliverable plans as only fluence optimization was performed (no segmentation). In that study, Erasmus-iCycle plans were used by a human planner to assist in the manual generation of deliverable plans with the clinical TPS. We observed that IMRT with 12 or more beams outperformed dual-arc VMAT in overall plan quality.

In this study, we built a system for fully automated generation of deliverable IMRT and VMAT plans for cervical patients. In this system, Erasmus-iCycle is used as a pre-optimizer for automated multi-criterial generation of a (non-deliverable) plan. The dose distribution of this plan is then automatically converted into a *patient-specific* template for automatic generation of a deliverable plan by Monaco. The building of this system for fully automated, multi-criterial generation of deliverable plans consisted of several steps and was done with plans of so-called tuning patients. In the first step, we further enhanced the Erasmus-iCycle plan quality for cervical cancer patients by optimizing the so-called wish-list (below) to better stress minimization of the small bowel V_45Gy_, as the most important OAR objective. Secondly, the automated building of patient-specific templates for automated plan generation by Monaco was tuned such that Erasmus-iCycle plans were properly re-constructed in Monaco. The generated patient-specific templates have a layering (Monaco terminology) determined by the Erasmus-iCycle priorities, in addition to parallel and serial cost functions with volume effect parameters for OARs (details in [9]).

Although Erasmus-iCycle is an algorithm for automated optimization of IMRT fluence profiles and selection of beam configurations [13], in this study, only beam profiles were optimized. To this purpose, Erasmus-iCycle uses the 2-phase ε-constraint (2pεc) lexicographic optimization algorithm for multi-criterial generation of a Pareto optimal IMRT plan. Plan generation with Erasmus-iCycle is based on a ‘*wish-list*’, which contains hard constraints that are strictly met, and prioritized objectives to steer the multi-criterial optimization [15,16]. The higher the priority of an objective, the higher the chance that the planning aim will be achieved, or even superseded. For each treatment site, the wish-list is generated in an iterative process where initial results are presented to the physician, and their feedback is used to adjust the wish-list, after which new plans are generated and presented to the physician. The result at the end of the process is a single wish-list, applicable to all patients of a certain treatment group.

The wish-list used in this study is presented in Table 1. The highest priority objective was given to the PTV, using the Logarithmic Tumor Control Probability (LTCP) function to obtain adequate PTV coverage for all plans (see [9,13] for details on the LTCP). The applied cell sensitivity parameter α was set to 0.8. To ensure steep dose fall-offs outside the PTV, as well as conformal dose distributions, one constraint and three objectives (priorities 2, 3 and 9) for shells around the PTV were defined. In line with clinical practice, SB had the highest priority objectives for OAR sparing (priorities 4, 5, 6 and 8 in Table 1). Priority 4, reduction of the SB equivalent uniform dose (EUD) with volume effect parameter 4 focusses on reduction of relatively high doses. A skin ring of 2 cm wide, from the body contour towards the patient’s internal, was defined to control entrance doses (priority 9). For sigmoid, colon, bladder and rectum, EUD and mean dose objectives were used as shown in Table 1.

**Table 1:** Wish-list applied for all automatic plan generations (autoVMAT and autoIMRT).

**Constraints**					
		**Volume**	**Type**	**Limit**	
		PTV	Max	105% of *D*^*p*^	
		PTV Shell 40 mm	Max	50% of *D*^*p*^	
		Unspecified Tissues	Max	105% of *D*^*p*^	
**Objectives**					
	**Priority**	**Volume**	**Type**	**Goal**	**Parameters**
	**1**	PTV	↓ LTCP	0.5	*D*^*p*^ = 46 Gy, α = 0.8
	**2**	PTV Shell 3 mm	↓ Max	95% of *D*^*p*^	
	**3**	PTV Shell 15 mm	↓ Max	75% of *D*^*p*^	
	**4**	Small Bowel	↓ EUD	45 Gy	k = 4
	**5**	Small Bowel	↓ EUD	15 Gy	k = 3
	**6**	Small Bowel	↓ Mean	10 Gy	
	**7**	Sigmoid	↓ EUD	30 Gy	k = 4
		Colon	↓ EUD	30 Gy	k = 4
	**8**	Small Bowel	↓ Max	*D*^*p*^	
	**9**	PTV Shell 25 mm	↓ Max	60% of *D*^*p*^	
		Skin Ring 20 mm	↓ Max	35% of *D*^*p*^	
	**10**	Bladder	↓ EUD	30 Gy	k = 4
	**11**	Rectum	↓ EUD	30 Gy	k = 4
	**12**	Sigmoid	↓ Mean	25 Gy	
		Colon	↓ Mean	25 Gy	
	**13**	Bladder	↓ Mean	25 Gy	
	**14**	Rectum	↓ Mean	25 Gy	

*Abbreviations*: *D*^*p*^ = prescribed dose, LTCP = Logarithmic Tumor Control Probability, EUD = Equivalent Uniform Dose, α = cell sensitivity, k = volume effect.

### Plan quality evaluations

Generated plans were visually checked and verified for clinical use by a clinician (STH or JWMM) and a medical physicist (MSH). For quantitative comparisons, autoVMAT, CLINICAL, manVMAT and autoIMRT plans were first normalized to obtain exactly equal median PTV doses and were then mutually compared regarding OAR sparing. For SB, the volume percentages receiving more than 45 Gy (V_45Gy_, the most relevant OAR plan parameter for clinical plan evaluation), and more than 15 Gy (V_15Gy_) (correlated to grade ≥ 3 acute SB toxicity [17]), and the mean dose, D_mean_, were analysed. For the other OARs, only D_mean_ was evaluated. Quantitative evaluation of plans also included the conformity index CI_95%_ (ratio of the total volume receiving 95% of the prescribed dose to the PTV) [18]. Paired two-sided Wilcoxon signed-rank tests were performed to compare the different planning strategies. Differences were considered statistically significant for *p* < 0.05.

### Delivery time comparisons

For a subgroup of 5 patients, autoVMAT and autoIMRT plans were generated for both MLCi2 and the fast Agility MLCs, and delivered at a treatment machine. The experiments for the Agility MLC were performed at the Dutch Cancer Institute, as this MLC was not available at Erasmus MC. A stopwatch was used to measure total delivery times (time elapsed from pushing the beam-on button till delivery of the last MU).

## Results

**Fig 1:**
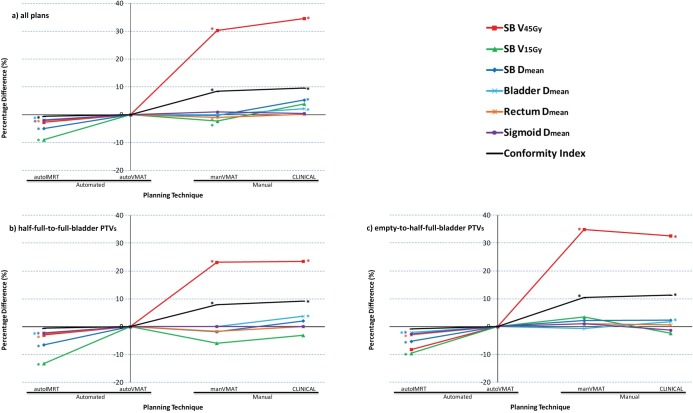
Percentage differences in mean OAR plan parameter values for all evaluated planning strategies, compared to autoVMAT. **(a) for all 44 plans, (b) and (c) for the 10 patients with large bladder filling induced PTV displacements.** Positive values point at an advantage for autoVMAT. ^*^: difference with autoVMAT is statistically significant. Average absolute values for autoVMAT: SB V_45Gy_ = 15.4%, SB V_15Gy_ = 68.7%, SB D_mean_ = 25.5 Gy, Bladder D_mean_ = 43.5 Gy, Rectum D_mean_ = 42.9 Gy, Sigmoid D_mean_ = 42.3 Gy and CI_95%_ = 1.174.

All generated plans were clinically acceptable and achieved adequate target coverage (i.e., at least 99.5% of the PTV received 95% of the prescribed dose), and PTV doses of more than 110% did not occur. After plan normalizations to obtain equal median PTV doses (M&M section), differences between corresponding autoVMAT, CLINICAL, manVMAT and autoIMRT plans in PTV V_95%_ were within 0.3%. Table 2 reports pair-wise comparisons of investigated planning strategies regarding OAR dose delivery. (Fig 1) shows for all planning strategies, mean differences in OAR plan evaluation parameters compared to autoVMAT.

**Table 2:** Pair-wise comparisons of planning strategies.

			autoIMRT			autoVMAT			manVMAT			CLINICAL	
					* *	*p-value*	*#Plans*	* *	*p-value*	*#Plans*	* *	*p-value*	*#Plans*
	**a**	** **				NS	(26 | 18)		<0.001	(44 | 00)		<0.001	(42 | 02)
	**b**	** **				<0.001	(37 | 07)		NS	(22 | 22)		<0.001	(31 | 12)
	**c**	** **				<0.001	(40 | 04)		NS	(26 | 18)		<0.001	(41 | 03)
**autoIMRT**	**d**	** **				<0.001	(42 | 02)		0.001	(33 | 11)		<0.001	(44 | 00)
	**e**	** **				<0.001	(41 | 03)		0.026	(29 | 15)		<0.001	(37 | 07)
	**f**	** **				<0.001	(29 | 02)		<0.001	(26 | 05)		0.002	(24 | 07)
	**g**	** **				0.018	(28 | 16)		<0.001	(44 | 00)		<0.001	(39 | 05)
	** **	** **											
	**a**	** **	0.3±1.5 (2.7%)						<0.001	(41 | 03)		<0.001	(43 | 01)
	**b**	** **	4.5±4.7 (9.0%)						0.011	(14 | 30)		NS	(26 | 18)
	**c**	** **	1.0±0.7 (4.9%)						NS	(16 | 28)		<0.001	(35 | 09)
**autoVMAT**	**d**	** **	0.8±0.5 (1.8%)						NS	(21 | 23)		<0.001	(39 | 05)
	**e**	** **	0.9±0.6 (2.1%)						0.041	(15 | 29)		NS	(26 | 18)
	**f**	** **	0.8±0.6 (2.0%)						NS	(18 | 13)		NS	(19 | 12)
	**g**	** **	0.01±0.02 (0.6%)						<0.001	(44 | 00)		<0.001	(39 | 05)
	** **	** **											
	**a**	** **	3.8±2.7 (32.7%)			3.5±3.0 (30.3%)						NS	(26 | 18)
	**b**	** **	1.5±11.6 (6.4%)			-2.9±10.4 (-2.3%)						0.037	(34 | 10)
	**c**	** **	0.8±2.3 (5.0%)			-0.3±2.1 (-0.2%)						0.003	(34 | 10)
**manVMAT**	**d**	** **	0.8±1.4 (1.8%)			0.0±1.3 (0.0%)						<0.001	(36 | 08)
	**e**	** **	0.5±1.2 (1.2%)			-0.4±1.3 (-0.9%)						0.006	(32 | 12)
	**f**	** **	1.1±0.9 (3.0%)			0.3±1.0 (1.0%)						NS	(14 | 17)
	**g**	** **	0.11±0.05 (8.9%)			0.10±0.05 (8.4%)						NS	(25 | 17)
	** **	** **											
	**a**	** **	4.7±4.2 (37.2%)			4.4±4.1 (34.6%)			0.9±3.7 (6.0%)				
	**b**	** **	7.3±11.5 (12.9%)			2.8±12.1 (3.9%)			5.8±15.9 (6.3%)				
	**c**	** **	2.4±1.7 (10.2%)			1.4±1.8 (5.3%)			1.7±3.0 (5.1%)				
**CLINICAL**	**d**	** **	1.7±1.0 (4.0%)			1.0±1.0 (2.2%)			1.0±1.4 (2.2%)				
	**e**	** **	1.0±1.2 (2.3%)			0.1±1.3 (0.2%)			0.5±1.3 (1.1%)				
	**f**	** **	0.8±1.3 (1.5%)			0.0±1.4 (0.5%)			-0.3±1.9 (-1.5%)				
	**g**	** **	0.13±0.11 (10.2%)			0.12±0.10 (9.7%)			0.02±0.10 (1.3%)				
			*Δmean±1SD (PD)*			*Δmean±1SD (PD)*			*Δmean±1SD (PD)*				

*Abbreviations*: NS = no statistically significant difference i.e. *p* > 0.05, SD = standard deviation, PD = percentage difference.

In each comparison (table cell), going from top to bottom, data refer to (a) SB V_45Gy_, (b) SB V_15Gy_, (c) SB D_mean_, (d) Bladder D_mean_, (e) Rectum D_mean_ (f) Sigmoid D_mean_, and (g) CI_95%_. Below the table diagonal, mean point differences with 1 SD, and mean relative percentage differences (between brackets) are shown, while *p* values and the numbers of plans with lowest OAR dose for one or the other strategy are presented above the diagonal; (n|m): for n plans, the strategy indicated at the vertical axis has lowest OAR dose, while for m plans the strategy mentioned at the horizontal axis is superior. Positive Δmean indicates that the strategy along the horizontal axis is superior (reduced OAR dose delivery).

### autoVMAT vs. Clinical plan quality

Automatically generated dual-arc VMAT plans showed superior plan quality compared to the CLINICAL plans (Table 2, Fig 1). For all OAR evaluation parameters, the mean values were on average lowest for autoVMAT, for 4/7 parameters the difference was statistically significant (*p*<0.001, Table 2). For 43/44 plans, SB V_45Gy_ was lowest with autoVMAT, and for 35/44, SB D_mean_ was lowest (Table 2).

### autoVMAT vs. manVMAT plan quality

In 41/44 cases, autoVMAT was lowest with respect to the most important OAR plan parameter, i.e. SB V_45Gy_ (*p*<0.001, average reduction 30.3%), while SB V_15Gy_ increased by on average 2.3% (*p* = 0.011), and SB D_mean_ by 0.2% (NS) (Table 2, Fig 1A). For the other OARs, differences were small (≤ 1%). All 44 autoVMAT plans had lower conformity index, CI_95%,_ than the corresponding manVMAT plan (*p*<0.001).

**Fig 2:**
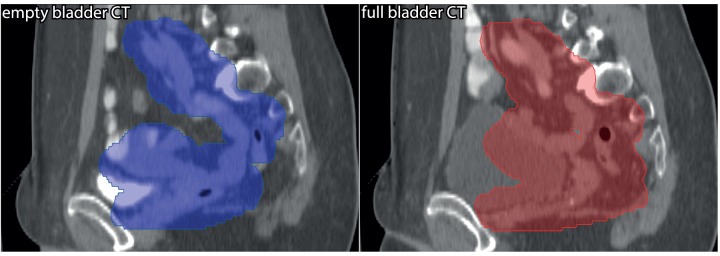
For a study patient, sagittal views of the empty bladder CT scan with the empty-to-half-full-bladder PTV (blue) projected on it (left), and of the full bladder CT scan with the half-full-to-full-bladder PTV (red) on the (right). The higher degree of concavity of the empty-to-half-full-bladder PTV increases the complexity of small bowel sparing in manual planning.

As illustrated in Fig 1B and 1C, for the large movers, the increase in plan quality with autoVMAT compared to manVMAT was most pronounced for PTVs for empty-to-half-full bladder. With autoVMAT, SB V_45Gy_ reduced on average by 34.9% (SD = 25.9%, range: [-0.5%, 92.7%]) for these PTVs, compared to 23.1% (SD = 15.7%, range: [-0.9%, 55.3%]) for the PTVs belonging to half-full-to-full bladder. For empty-to-half-full bladder PTVs, SB V_15Gy_ was on average reduced by 3.4% using autoVMAT instead of manVMAT, while there was an increase by 5.9% for half-full-to-full bladder PTVs. With autoVMAT, SB D_mean_ was 2.2% lower for empty-to-half-full bladder PTVs, while for half-full-to-full bladder PTVs an average increase of 1.8% was observed. The enhanced positive impact of autoVMAT for empty-to-half-full bladder PTVs is attributed to the higher degree of concavity of these PTVs (see Fig 2 for example), making it relatively difficult for the human planner to optimally spare the SB in the manVMAT plan.

### autoVMAT vs. autoIMRT plan quality

Compared to autoVMAT, in automatically generated 20-beam equi-angular IMRT plans (autoIMRT) SB V_45Gy_ reduced by 2.7%, while also resulting in a 9.0% reduction in SB V_15Gy_, and a reduction of 4.9% in SB D_mean_ (*p*<0.001, Table 2). Differences in bladder, rectal and sigmoid doses were small, but all in favor of autoIMRT (Fig 1).

### Planning times

To maximally challenge the developed procedure for automated plan generation (autoVMAT and autoIMRT) regarding plan quality, the expert planner was allowed as much time as needed for the manual generation of high-quality manVMAT plans. Generation of the latter took on average 6 h, including 3 h of hands-on planning time and Monte Carlo dose calculations. For the manual generation of the CLINICAL plans, planning times were not recorded. We estimate that these times were around 2.5 hours on average.

For fully automatic plan generation, Erasmus-iCycle optimizations took on average 3 h calculation time, and the automatic reconstruction of a clinically deliverable plan in Monaco took on average 4.5 h for autoVMAT and 2.5 h for autoIMRT. Optimizations for automated plan generation were often performed in evenings or during the night.

### Treatment time comparisons

Table 3 shows for a subgroup of 5 patients measured treatment times for the generated autoVMAT and autoIMRT plans. Compared to MLCi2, the Agility MLC exhibits treatment time reductions of ~ 30% and 42% for autoVMAT, and autoIMRT, respectively, while preserving the same plan quality. With Agility, mean treatment times for delivery of autoVMAT and autoIMRT plans were 2.9 and 9.1 min.

**Table 3:** Measured treatment delivery times for autoVMAT and autoIMRT using the MLCi2 and Agility MLCs.

Planning Technique	Patient	MLCi2 Treatment Delivery Time (min)	Agility Treatment Delivery Time (min)	Difference (min/%)
**autoVMAT**	Pt 1	4.5	2.9	1.6 / 35.6
	Pt 2	3.8	2.7	1.1 / 28.9
	Pt 3	4.1	3.2	0.9 / 22.0
	Pt 4	4.4	3.1	1.3 / 29.5
	Pt 5	3.9	2.6	1.3 / 33.3
	**Mean**	**4.1**	**2.9**	**1.2 / 29.9**
**autoIMRT**	Pt 1	15.0	9.8	5.2 / 34.7
	Pt 2	17.6	9.5	8.1 / 46.0
	Pt 3	14.0	7.9	6.1 / 43.6
	Pt 4	17.5	10.4	7.1 / 40.6
	Pt 5	14.1	7.8	6.3 / 44.7
	**Mean**	**15.6**	**9.1**	**6.6 / 41.9**

## Discussion

We have developed and validated a fully automated VMAT treatment planning solution for plan library filling in adaptive radiotherapy for cervical cancer patients. Based on the positive findings presented here, the method is now in routine clinical use. Compared to the previous use of manually generated, 9-beam IMRT plans, the automation rendered hands-on planning obsolete and resulted in higher quality dose distributions. Due to the switch to VMAT, the daily treatment time was also reduced, reducing the negative impact of time-dependent intra-fraction bladder filling and changes in patient setup [19]. We demonstrated that autoVMAT plans were also of higher quality than VMAT plans manually generated by an expert planner in the absence of time pressure. For autoVMAT, SB V_45Gy_ was reduced by 30.3% on average while SB V_15Gy_ increased by 2.3%. This is related to the fact that, according to the applied wish-list in this study, minimization of the dose in the small bowel at the 45 Gy level is given higher planning priority than at the 15 Gy level, and this likely reduces grade ≥ 3 acute SB toxicity [17]. The benchmark with automatically generated 20-beam equi-angular IMRT plans (autoIMRT) demonstrated a superior plan quality compared to autoVMAT. However, with the currently available MLCi2 multi-leaf collimators in our department, the treatment delivery time would increase from 4.1 min for autoVMAT to 15.6 min for autoIMRT. This increase led to the departmental decision to always treat with autoVMAT in order to limit the impact of intra-fraction bladder filling and patient motion, and to increase patient throughput. However, the experiments with a much faster Agility MLC, performed in a different institution, showed delivery times of 2.9 and 9.1 min for autoVMAT and autoIMRT, respectively. With 9.1 min, autoIMRT might be the treatment modality of choice for selected patients with a high plan quality gain compared to autoVMAT, in case of availability of an Agility MLC.

Until recently, plan libraries for cervical cancer patients always contained a 3DCRT back-up plan, to be applied in case of an occasional daily dosimetric mismatch between the VMAT plan(s) in the library and the anatomy-of-the-day, or in case of poor CBCT image quality (see Introduction section). To further exploit the possibility of automated planning, we have now replaced the manually generated 3DCRT plan by an automatically generated VMAT plan with a generous CTV-PTV margin, to be used as back-up plan. The reduction of workload related to automated planning does also allow extension of the pre-treatment established, patient-specific libraries with more plans, each with a smaller CTV-PTV margin. This extension is currently being investigated, as well as extension based on daily acquired CBCTs.

In this paper, we investigated automated planning for patients that did not have involvement of pelvic or para-aortic lymph nodes. For these patients, only the pelvic nodes (superior field border at the level of the aortic bifurcation) are irradiated to the prescribed dose of 46 Gy, delivered in 23 fractions. For locally advanced cervical cancer patients with positive pelvic or para-aortic lymph nodes, toxicity can be substantial due to enlarged fields in superior direction and increased (boost) dose, while the dose that can be given is restricted by tolerances of the surrounding OARs such as kidneys, spinal cord, and small bowel. This can increase the complexity of treatment planning, requiring an enhanced workload effort, as well as skills and experience of the planner. We believe that also for these challenging patients, automated planning holds a strong promise for treatment plan improvement, compared to the current manual planning. However, involved investigations were outside the scope of this paper. To the best of our knowledge, this is the first paper on fully automated, multi-criterial generation of clinically deliverable plans for cervical cancer. It is also the first paper on filling of libraries with automatically generated, clinically deliverable plans, used in plan-of-the-day radiotherapy. In a previous study, [9] Voet et al. developed a system for fully automatic VMAT plan generation for prostate cancer patients, based on an Erasmus-iCycle pre-optimization and final plan generation in Monaco. In the current study we adapted this method for fully automatic, multi-criterial generation of deliverable plans for cervical cancer patients, both for VMAT and for static gantry IMRT. Recently, [20,21] Quan et al. developed an automatic inverse planning algorithm for dual-arc VMAT and IMRT for prostate and lung. The initial objectives in their work were defined on the basis of previous planning experience. Their ‘autoplans’ were consistently better, or no worse, than the manual plans in terms of tumor coverage and normal tissue sparing [22]. In another study Wu et al. [23] developed a method using overlap volume histograms and IMRT data to guide and automate head and neck VMAT planning. In terms of PTV coverage and OAR sparing their automatically IMRT-data driven VMAT plans were comparable to clinical IMRT plans. A large and rather mature IMRT plan dataset was required to effectively guide VMAT planning. In most recent studies [24,25] a comprehensive knowledge-based method was used for predicting achievable dose-volume histograms (DVHs) to standardize and improve treatment planning. [25] Tol et al. evaluated the method for head and neck cancer using a library of different patient plans to make a model that can predict achievable DVHs for defining optimization objectives for new patients. The performance of the proposed method depended highly on the geometrical characteristics of the model library and the quality of the IMRT plan database. Automated plan generation with Erasmus-iCycle, as described in [13,15,16] and briefly summarized above, does not depend on a database with DVHs of previously treated patients. Instead, the generation of the tumor site specific wish-lists, containing hard constraints and planning objectives with priorities, results in a generalized approach of knowledge-based planning, using previous planning experience as well as new insights for example obtained from clinical studies or literature. The quality of automatically generated new plans does not depend on the quality of the plans in a database. Moreover, in case of an adaptation in the clinical protocol, automatic plan generation does only require an adaptation of the wish-list, and does not have to wait until a new database with manually generated, high-quality plans has been produced first.

## Conclusions

A method for fully automated VMAT treatment plan generation for filling of plan libraries in adaptive radiotherapy for advanced cervical cancer has been developed and validated. Quality of automatically generated VMAT plans was superior to manually generated plans, while planning workload was fully avoided. Based on the study results, automatic VMAT plan generation has been implemented in clinical routine. Due to the absence of planning workload, extension of plan libraries to enhance OAR sparing has become feasible.

## Supporting Information

S1 File The data underlying the findings in present study. (XLSX)
